# P-1832. Closing the Care Gap: Community-Based Strategies Linking Patients with HCV and Substance Use Disorder in Appalachia

**DOI:** 10.1093/ofid/ofaf695.2001

**Published:** 2026-01-11

**Authors:** Merly Konathapally, Sarah Henrickson Parker, Mariana Gomez de la Espriella

**Affiliations:** Mary Washington Healthcare, Fredericksburg, VA; Virginia Tech Carilion School of Medicine, Roanoke, Virginia; Carilion Clinic, Virginia Tech, Salem, Virginia

## Abstract

**Background:**

In 2020, the rate of Hepatitis C Virus (HCV) infection in Roanoke City, was three times higher than the Virginia state rate. Despite advances in treatment, significant barriers remain in linkage-to-care (LTC), especially in patients with substance use disorder (SUD). Previous studies suggest that addressing costs and providing patient navigation can improve LTC following HCV diagnosis for patients with SUD.

**Table 1.**
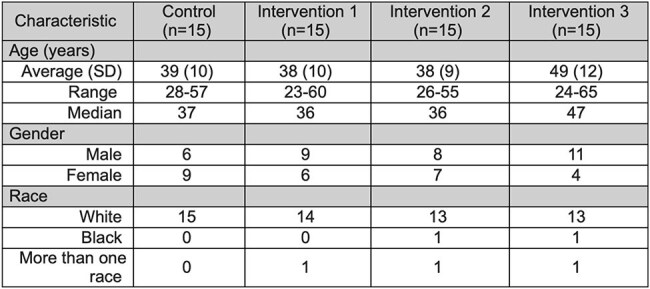
Demographic characteristics of control, Intervention 1, Intervention 2, and Intervention 3 group participants.

**Figure 1.**
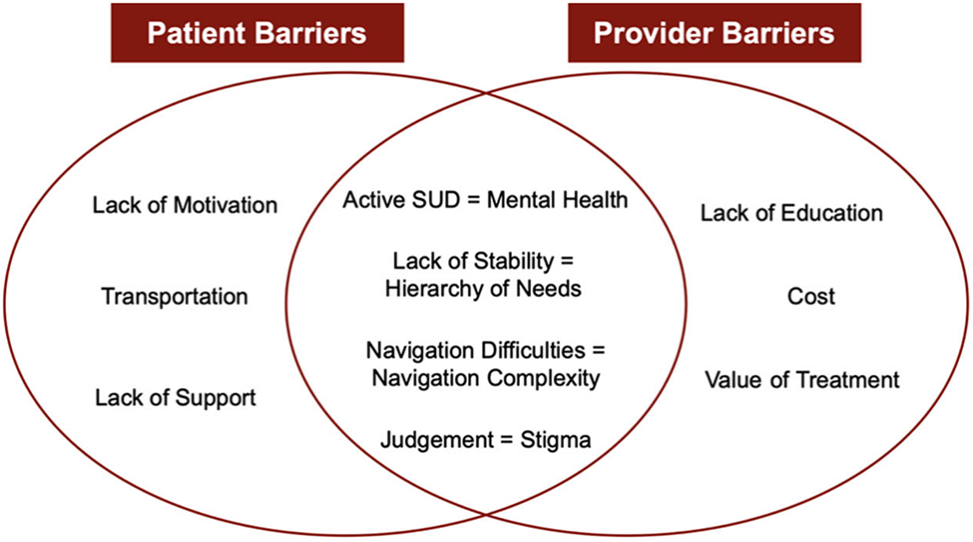
Comparison between subthemes of patient-identified and provider-identified barriers to care. Overlapping subthemes are indicated with an equal sign, where patient subtheme = provider subtheme. SUD = Substance Use Disorder.

**Methods:**

The study was conducted in two phases. In the qualitative phase, interviews from ten patients and ten health care providers were coded for themes related to barriers and facilitators to care. In the quantitative phase, a pragmatic trial approach was used to evaluate real-world interventions across four groups from July 2022 to January 2025 (Table 1). Fifteen patients in the control group were scheduled for an HCV treatment appointment and connected with peer recovery specialists to facilitate LTC. In Intervention 1, 15 patients received the same standard of care and vouchers prior to appointment. In Intervention 2, 15 patients were provided vouchers at the time of appointment as an incentive. In Intervention 3, 15 patients received vouchers throughout the HCV care continuum, participated in telehealth visits, and received peer navigation through a local Harm Reduction program.

**Figure 2.**
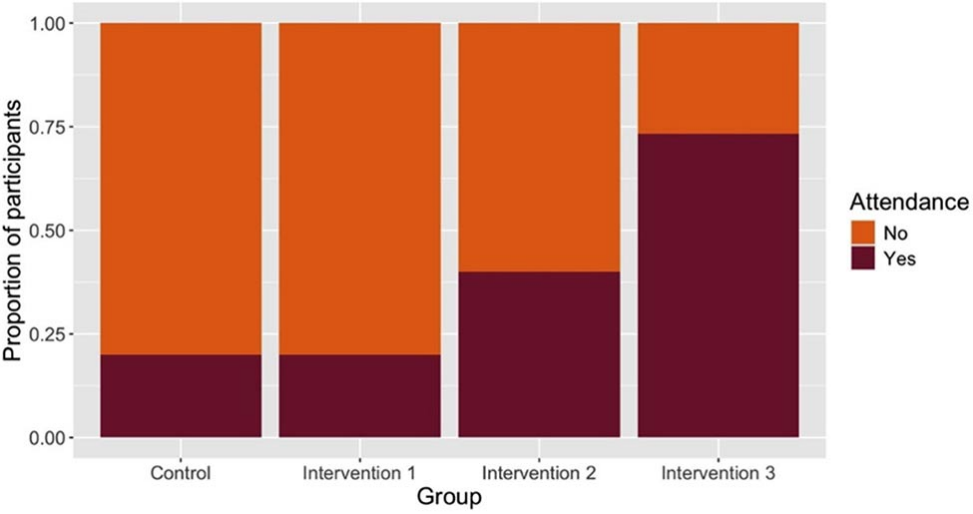
Proportion of patients attending first treatment visit for control, Intervention 1, Intervention 2, and Intervention 3 groups. Yes = patient attended first treatment visit, No = patient did not attend first treatment visit.

**Results:**

Analysis of interview responses using grounded theory has identified barriers to care, including transportation, phone, and housing access. Patient and provider responses show similarities in the complexity of HCV treatment (Figure 1). Comparison of the control and intervention groups shows a statistical difference in attendance at the first treatment visit, χ2(3, N = 60) = 12.06, *p* = .007, specifically from Intervention 3 (Figure 2).

**Conclusion:**

The interview themes demonstrate that HCV treatment is a complex process, and patient navigator services are considered critical in LTC. The combination of vouchers, peer navigation, telehealth and community partnership in Intervention 3 significantly improved attendance at the first treatment visit, though the study is limited in distinguishing which component improved LTC. Future studies may explore additional ways to improve LTC, including street medicine and inpatient treatment initiation.

**Disclosures:**

All Authors: No reported disclosures

